# Heat tolerance limits of Mediterranean songbirds and their current and future vulnerabilities to temperature extremes

**DOI:** 10.1242/jeb.244848

**Published:** 2022-12-09

**Authors:** Julián Cabello-Vergel, Erick González-Medina, Manuel Parejo, José M. Abad-Gómez, Núria Playà-Montmany, Daniel Patón, Juan M. Sánchez-Guzmán, José A. Masero, Jorge S. Gutiérrez, Auxiliadora Villegas

**Affiliations:** ^1^ Conservation Biology Research Group, Faculty of Sciences, University of Extremadura, 06006 Badajoz, Spain; ^2^ Ecology Department, Faculty of Sciences, University of Extremadura, 06006 Badajoz, Spain; ^3^ Ecology in the Anthropocene, Associated Unit CSIC-UEX, Faculty of Sciences, University of Extremadura, 06006 Badajoz, Spain

**Keywords:** Climate warming, Heat stress, Heatwaves, Passerines, Thermal physiology, Thermoregulatory traits

## Abstract

Songbirds are one of the groups most vulnerable to extreme heat events. Although several recent studies have assessed their physiological responses to heat, most of them have focused solely on arid-zone species. We investigated thermoregulatory responses to heat in eight small-sized songbirds occurring in the Mediterranean Basin, where heatwaves are becoming more frequent and intense. Specifically, we determined their heat tolerance limits (HTLs) and evaporative cooling efficiency, and evaluated their current and future vulnerabilities to heat in southwestern Iberia, a Mediterranean climate warming hotspot. To do this, we exposed birds to an increasing profile of air temperatures (*T*_a_) and measured resting metabolic rate (RMR), evaporative water loss (EWL), evaporative cooling efficiency (the ratio between evaporative heat loss and metabolic heat production) and body temperature (*T*_b_). HTL ranged between 40 and 46°C across species, and all species showed rapid increases in RMR, EWL and *T*_b_ in response to increasing *T*_a_. However, only the crested lark (*Galerida cristata*) achieved an evaporative cooling efficiency greater than 1. The studied songbirds currently experience summer *T*_a_ maxima that surpass the upper critical temperatures of their thermoneutral zone and even their HTL. Our estimates indicate that five of the eight species will experience moderate risk of lethal dehydration by the end of the century. We argue that the limited heat tolerance and evaporative cooling efficiency of small-sized Mediterranean songbirds make them particularly vulnerable to heatwaves, which will be exacerbated under future climate change scenarios.

## Teaser

**Highlighted Article:** Mediterranean songbirds have limited cooling efficiencies and heat tolerance limits. This makes them sensitive to climate warming, particularly so under future climate change scenarios.

## INTRODUCTION

Earth's climate is warming at an unprecedented rate, pushing many species toward and beyond the upper temperatures at which they can survive (Quintero and Wiens, 2013; Riddell et al., 2019; IPCC, 2021). Besides global increases in mean surface air temperatures, climate warming is expected to bring more frequent, intense and long-lasting extreme climatic events, such as heatwaves (Coumou and Robinson, 2013; IPCC, 2021). Temperature extremes associated with heatwaves can challenge the physiological capacities of endotherms to maintain their thermal balance, resulting in adverse effects on their fitness (reviewed by Cunningham et al., 2021). These effects can be either sub-lethal (Du Plessis et al., 2012; Cunningham et al., 2013; van de Ven et al., 2019, 2020) or lethal, sometimes resulting in mass mortality events across wide geographical areas, from deserts to circumpolar regions (see Welbergen et al., 2008; Saunders et al., 2011; McKechnie et al., 2012, 2021a; Quintana et al., 2022).

Among endotherms, small songbirds (order Passeriformes) are especially vulnerable to heatwaves owing to their diurnal habits, small body sizes with large surface area to volume ratios, and high metabolic rates (Albright et al., 2017). Panting is the main physiological response of songbirds to maintain thermal balance and avoid lethal hyperthermia at high ambient temperatures, yet it is expensive in terms of energy and water (Calder and Schmidt-Nielsen, 1966; Dawson, 1982; Wolf and Walsberg, 1996; McNab, 2002). This is one of the reasons why songbirds generally achieve lower evaporative cooling efficiencies – defined as the quotient between evaporative heat loss (EHL) and metabolic heat production (MHP) – than other bird groups that rely on more efficient physiological mechanisms, such as gular fluttering or cutaneous evaporative water loss (reviewed in McKechnie et al., 2021a,b).

In this context, the study of heat tolerance limits (HTLs, i.e. maximum air temperature tolerated before the onset of severe heat stress) is essential to determine the vulnerability of species to global warming (Williams et al., 2008). Over the last decade, an increasing number of studies have focused on avian thermoregulation in the heat, particularly in songbirds (e.g. Whitfield et al., 2015; McKechnie et al., 2017; Smith et al., 2017; Oswald et al., 2018b; Smit et al., 2018; Kemp and McKechnie, 2019; Czenze et al., 2020; Pollock et al., 2020; O'Connor et al., 2021; Freeman et al., 2022), yet the majority of them have focused on desert and arid-zone species (e.g. Whitfield et al., 2015; McKechnie et al., 2017; Smith et al., 2017; Kemp and McKechnie, 2019; Czenze et al., 2020). In fact, studies on HTL and evaporative cooling capacity of species from tropical, temperate and subpolar areas are very scarce (Oswald et al., 2018b; Pollock et al., 2020; O'Connor et al., 2021; Freeman et al., 2022), particularly in the Mediterranean region (Playà-Montmany et al., 2021). These studies have revealed a within-order biogeographic variation in heat tolerance, with desert and arid-zone species dealing better with heat stress by achieving greater HTLs and evaporative cooling efficiencies (see Whitfield et al., 2015; McKechnie et al., 2017; Smith et al., 2017; Kemp and McKechnie, 2019; Czenze et al., 2020) than those from tropical, temperate or subpolar regions (Pollock et al., 2020; O'Connor et al., 2021).

The Mediterranean Basin is warming faster than other regions across the globe (IPCC, 2021), having experienced a 1.5°C increase in mean annual surface temperature since preindustrial values. Indeed, climate change scenarios predict increments of 1.8–3.7°C (SSP2-4.5 and SSP5-8.5, respectively) as well as more frequent and intense heatwaves to the end of this century (Gutiérrez et al., 2021). Within the Mediterranean Basin, Southwestern Iberia is predicted to be one of the more sensitive areas to global warming effects, with studies predicting a twofold increase in the number, frequency and severity of heatwaves (Cardoso-Pereira et al., 2017). Understanding how Mediterranean species differ in their HTLs, therefore, is critical for accurately forecasting their vulnerability under future climate change prospects. As HTLs well exceed the upper critical temperature (*T*_uc_) of the thermoneutral zone, they are more valuable to inform vulnerability predictive models (see Albright et al., 2017; Conradie et al., 2019, 2020) than *T*_uc_ (Khaliq et al., 2014; Mitchell et al., 2018).

In this study, we evaluated the HTLs and evaporative cooling efficiency of small-sized (from ∼10 to ∼34 g) Mediterranean resident songbirds. Moreover, we investigated current and future vulnerability of the species to heat extremes under different climate warming scenarios, using several proxies: (1) the number of days above *T*_uc_ (in which birds are forced to actively thermoregulate to maintain thermal balance), (2) the number of days above HTL (days in which birds face direct risk of lethal hyperthermia) and (3) lethal dehydration risks during the hottest days. We predicted that Mediterranean songbirds would display lower HTLs and evaporative cooling efficiencies than arid-zone and desert songbirds previously studied, but in the range found in those from tropical and northern-temperate climates. Based on previous findings (reviewed by McKechnie et al., 2021b), we expected that larger species would deal better with heat than smaller ones. Finally, we also predicted that summer maximum temperatures experienced by the studied species (often exceeding 40°C) may currently compromise their physiological thermoregulatory capabilities, which would be exacerbated under future climate warming scenarios.

**List of symbols and abbreviations**

EHL: evaporative heat loss
EWL: evaporative water loss
HTL: heat tolerance limit
*M*_b_
: body mass
MHP: metabolic heat production
PIT: passive integrated transponder
RMR: resting metabolic rate
*T*_a_
: air temperature
*T*_b_
: body temperature
*T*_uc_
: upper critical temperature
*V̇*_O_2__
: rate of oxygen consumption

## MATERIALS AND METHODS

### Study site

The study took place in Badajoz, southwestern Spain (Fig. S1), during the summers of 2020 and 2021 (from late June to early September). The climate in the study area is classified as Mediterranean, with mild winters and warm and dry summers. Maximum air temperatures in summer average 31.8±2.1°C (mean±s.d.), with recent records of up to 46°C (State Meteorology Agency, https://opendata.aemet.es/). During the last 30 years, this area has presented a significant increase in the number of hot days per summer, currently experiencing up to 50 days in which maximum ambient temperature exceeds 35°C (Fig. S2).

There are three main types of habitat in our study area: (i) Mediterranean forest, composed of different *Quercus* species with dense shrub cover under the canopy; (ii) dehesas (transformation of the original Mediterranean forest by clearance and brushwood removal, providing a typical landscape of savannah-like open woodland with scattered trees); and (iii) farmlands, open landscapes dominated by cereal lands, olive groves, vineyards and fallows.

### Bird species and capture

We measured HTL and evaporative cooling capacity in eight resident Palearctic songbirds widely distributed across the Mediterranean Basin: crested lark [*Galerida cristata* (Linnaeus 1758), Alaudidae; *N*=6], house sparrow [*Passer domesticus* (Linnaeus 1758), Passeridae; *N*=18], Spanish sparrow [*Passer hispaniolensis* (Temminck 1820), Passeridae; *N*=15], chaffinch (*Fringilla coelebs* Linnaeus 1758, Fringillidae; *N*=21), greenfinch [*Chloris chloris* (Linnaeus 1758), Fringillidae; *N*=20], goldfinch [*Carduelis carduelis* (Linnaeus 1758), Fringillidae; *N*=17], serin [*Serinus serinus* (Linnaeus 1766), Fringillidae; *N*=11] and great tit (*Parus major* Linnaeus 1758, Paridae; *N*=16).

We captured adult and fully developed first calendar year individuals by mist-netting. Adults moulting wings and tail feathers, or showing heavy body moult, were discarded from heat tolerance trials, as moulting is energetically expensive (e.g. Delhey et al., 2020) and could influence metabolic rates (e.g. Klaassen, 1995). All summer juveniles used in measurements were non-moulting individuals. Upon capture, birds were transferred to cloth bags and then moved quickly to large indoor aviaries (320×260×255 cm) at the University of Extremadura (Badajoz), where they were kept with food and water *ad libitum* until heat tolerance trials were performed. Individuals spent a maximum of 24 h in captivity and were always released at the site of capture. All experimental procedures were approved by the bioethical committee of the University of Extremadura (76/2018) and performed under governmental licenses CN0002/20/ACA and CN0002/21/ACA.

### Body temperature and gas exchange measures

We used temperature-sensitive passive integrated transponder (PIT) tags (BioTherm13 13×2.12 mm, Biomark, USA) to monitor subcutaneous body temperature during heat tolerance trials. Recent studies have shown that subcutaneous body temperature is close to core body temperature at high *T*_a_ (e.g. Oswald et al., 2018a; O'Connor et al., 2021 supplementary information); thus, we used subcutaneous body temperature as an indicator of body temperature (hereafter *T*_b_). Tags were inserted under the skin in the interclavicular region, as intraperitoneal implantation has shown adverse effects in small-sized birds (<25 g) (Oswald et al., 2018a). To prevent PIT tag loss during the trials, we employed approach stitches to seal the wound. *T*_b_ readings were recorded every 5 s during heat tolerance trials by a racket antenna (model F201F-ISO, Biomark) connected to an external reader (IS1001 Multiplexing Transceiver System, Biomark).

We measured oxygen consumption (*V̇*_O_2__) and EWL (*V̇*_H_2_O_) using an open flow-through respirometry system. Birds were placed individually in a 4.9 liter plastic chamber set inside a temperature-controlled cabinet (model F-4, Ibercex) that allowed us to adjust *T*_a_. Within the chamber, a wire mesh platform was placed 4 cm above a 1 cm mineral oil layer to trap excreta and allow birds to perch. *T*_a_ inside the chamber was continuously measured using a calibrated thermistor probe (TC-100, Sable Systems).

Ambient dried air was supplied by a compressor (model R-110300, MESTRA), and the air stream was split into two channels: the baseline (an empty chamber) and the metabolic chamber. Flow rates to each channel were controlled by mass flow controllers (MFS5, Sable Systems) or a mass flow generator (Flowkit, Sable Systems) when flow rates greater than 3000 ml min^−1^ were needed. The flow rate to the baseline was always set to 1000 ml min^−1^, while flow rates to the metabolic chamber were adjusted (2000–9000 ml min^−1^) during the trials to maintain low ambient humidity values (<1 kPa) at the same time that oxygen depletion rates remained detectable. This permitted birds to stay calm and maintain water vapor gradients that did not potentially preclude EWL rates (see van Dyk et al., 2019). We sequentially switched manually between the baseline and metabolic chambers using a multiplexer (RM-8, Sable Systems). Excurrent air was subsampled at a rate of 200 ml min^−1^ (SS3 Subsampler, Sable Systems) and pulled sequentially through an H_2_O analyzer (RH300 model, Sable Systems), a Drierite^®^ column and a O_2_ analyzer (FC-10 model, Sable Systems). Both analyzers were periodically zeroed and spanned. An analog to digital converter (UI2 model, Sable Systems) was used to digitalize voltage outputs from the analyzers and the thermistor probe, and Expedata software (version 1.9.14, Sable Systems) was employed to record these outputs with a sampling rate of 1 s.

### Experimental protocol

Heat tolerance trials took place during the birds' active phase, and a single bird was measured at a time. Food was withdrawn at least 2 h before trials started, so we assumed that birds were in a post-absorptive state. Gas exchange rates were measured along a stepped profile of increasing *T*_a_: 30, 33, 37 and 40°C, and 2°C increments at *T*_a_>40°C until HTL was reached, following a similar procedure employed in recent studies (e.g. Whitfield et al., 2015). Recent work has demonstrated that stepped protocols yield similar results to steady-state protocols that exposed birds to each temperature during several hours, which reduces the time that birds are confined in the metabolic chamber (Short et al., 2022). At the beginning of each trial, 10 min of baseline air was subsampled before switching to the metabolic chamber. The first *T*_a_ was maintained for approximately 30 min until stable traces of O_2_ and H_2_O and normothermic *T*_b_ values (40–42°C) were achieved. Then, the next *T*_a_ was sequentially set, until the end of the trial. Between successive target *T*_a_ and again at the end, 5 min of baseline were collected. Birds spent a minimum of 10 min at each target *T*_a_, and trials took no longer than 3 h. During measurements, we continuously monitored the bird’s behaviour with an infrared video camera.

Trials ended when birds reached their HTL, which occurred when birds started to show continuous active escape behaviour (pecking the walls, flapping or jumping around) or signs of severe heat stress, such as loss of righting response or balance, sudden decreases in oxygen consumption or EWL, and/or sustained uncontrolled increases (>0.1°C min^−1^) in *T*_b_ approaching or exceeding 45°C (following Whitfield et al., 2015, O'Connor et al., 2021). Following Smith et al. (2017), the birds' behaviour was scored from 0 to 5, with 0 corresponding to a completely calm bird and 5 to a bird showing sustained escape behaviour. For data analyses, highly active birds (scores 4–5) were not considered. When a bird reached its HTL, it was rapidly removed from the chamber and placed in front of a fan. We soaked its legs in alcohol to aid heat dissipation and monitored the bird until its *T*_b_ returned to normothermic values. Finally, we hydrated and returned it to the indoor aviaries. PIT tags were removed before releasing the birds.

### Data analyses

We corrected for O_2_ and H_2_O drift and lag in Expedata software and used eqns 10.2 and 10.9 from Lighton (2008) to calculate *V̇*_O_2__ and EWL, assuming a respiratory exchange ratio of 0.71 (birds in post-absorptive state during the metabolic trials) (Walsberg and Wolf, 1995). Resting metabolic rate (RMR) and EWL were extracted from the lowest 5 min values at each target *T*_a_ to which birds were exposed. RMR was converted to MHP (W) by multiplying *V̇*_O_2__ (ml min^−1^) by its oxyjoule equivalent, which was obtained from eqn 9.13 of Lighton (2018) assuming a respiratory quotient of 0.71 (i.e. metabolism consists solely of lipids). EWL was converted to EHL (W) assuming a latent heat of vaporization of 2.406 J mg^−1^ (Tracy et al., 2010). *T*_b_ was calculated as the average value during the final 10 min of exposure to each *T*_a_. We reported whole-animal RMR (W) and EWL values (mg h^−1^), although we also showed mass-specific slopes of both traits to favour interspecific comparisons.

All statistical analyses were performed in R (https://www.r-project.org/). To evaluate HTL and evaporative cooling efficiency, we determined inflection points for RMR, EWL, EHL/MHP and *T*_b_. We fitted broken-stick linear regressions for each species using the package segmented (Muggeo, 2009). Then, we adjusted generalized linear mixed-effect models using the package lme4 (Bates et al., 2015) to estimate the slopes of each variable above its inflection point in response to *T*_a_, including individual identity as a random factor as we had measurements for each bird at various *T*_a_. Initially, we included *M*_b_ as a covariate in all models, but as it did not emerge as significant nor improve model fit, we discarded it from intraspecific analyses.

Additionally, we tested for possible interspecific differences in thermoregulatory traits across Mediterranean songbirds. Initially, we fitted phylogenetic generalized least squares models using the package caper (https://CRAN.R-project.org/package=caper) to test for possible phylogenetic relatedness in thermoregulatory traits among our species. However, lambda showed values equal to zero for each trait tested (indicating an absence of phylogenetic effect; see Table S1). Therefore, we fitted generalized linear models with different thermoregulatory traits – mass-specific RMR, mass-specific EWL, EHL/MHP and *T*_b_ slopes above inflection points, evaporative scope (the ratio between maximum and minimum EWL), maximum EHL/MHP, and HTL – as response variables and mean *M*_b_ of each species (as a proxy of body size) as a predictor variable, to test for possible scaling of these thermal traits with body size. In the case of HTL, in addition to mean *M*_b_, we included mean evaporative scope as a predictor, as a previous study found that species with greater evaporative scopes achieve higher HTLs (Czenze et al., 2020).

### Vulnerability to high air temperatures

We evaluated current and future vulnerability to high temperatures of small Mediterranean resident songbirds across Extremadura, southwestern Spain. Previous work has forecasted particularly severe future increases in heatwaves in this region (Cardoso-Pereira et al., 2017; Viceto et al., 2019). To do so, we employed two metrics: the number of days in which maximum environmental temperature surpasses *T*_uc_ (i.e. a proxy of possible chronic deleterious effects of heat exposure on individual fitness), and the number of days in which maximum environmental temperature exceeds HTL (i.e. a proxy of lethal hyperthermia risk).

We obtained maximum temperature values on a daily basis from June to September, both at current times (2006–2021) and at the end of the century (2070–2100) under two different climate change scenarios (RCP4.5 and RCP8.5 in the framework of the Fifth Assessment Report of the IPCC; IPCC, 2014). RCP4.5 corresponds to a stabilization scenario that forecasts an additional increase in mean surface temperature of 1.8°C, while RCP8.5 represent a ‘business as usual’ scenario (unmitigated greenhouse gas emissions) and forecasts an additional increase of 3.7°C (IPCC, 2014). We obtained climate data projections for Extremadura from the Spanish Climate Change Office project AdapteCCa (https://adaptecca.es/). This data contained daily maximum temperature predictions from 16 different regional climate models (spatial resolution of 0.11 deg; EUR-11, ∼12.5 km) from EURO-CORDEX (Jacob et al., 2014) (see Supplementary Materials and Methods). Previous work has shown that these models have similar sensitivity and yield faithful predictions with respect to the reference period 1986-2005 (Barredo et al., 2018). We averaged daily model outputs to obtain the average number of days per summer above *T*_uc_ and above HTL for each species at different times and scenarios across each grid, and then we mapped these results (as in Conradie et al., 2019).

Furthermore, we calculated current and future lethal dehydration risks of Mediterranean songbirds during heatwaves following an approach similar to that of Albright et al. (2017). We assumed that birds retreat to shaded microhabitats and cease foraging during the hottest part of the day, thus impeding the replenishment of body water lost through evaporative means. After determining the *T*_a_ at which each species started to increase EWL, we used their slopes above the inflection point to calculate hourly EWL rates and hence the time needed to achieve their lethal dehydration thresholds (intended as cumulative EWL surpassing 15% of species *M*_b_; Albright et al., 2017) during extremely hot days (see Supplementary Materials and Methods for details). We estimated the total evaporative heat losses of species between 10:00 and 20:00 h, a period during which *T*_a_ is above the EWL inflection point for all the species considered during extremely hot days in the study area. Following Albright et al. (2017), we considered moderate risks of lethal dehydration when birds take ≤5 h to lose 15% of their *M*_b_, and severe risk when this threshold was met in ≤3 h.

Following previous studies (Albright et al., 2017; Cook et al., 2020), we predicted species-specific current risk of dehydration, by averaging hourly values of environmental temperature from the hottest 10 days during the last 20 years in the study area (State Agency of Meteorology, https://opendata.aemet.es/) (Fig. S3). To this hourly profile, we added the predicted temperature increase in mean surface temperature by the two climate change scenarios (RCP4.5 and RCP8.5) in the long term (2070–2100) (Fig. S3).

## RESULTS

### Resting metabolic rate

**Fig. 1.**
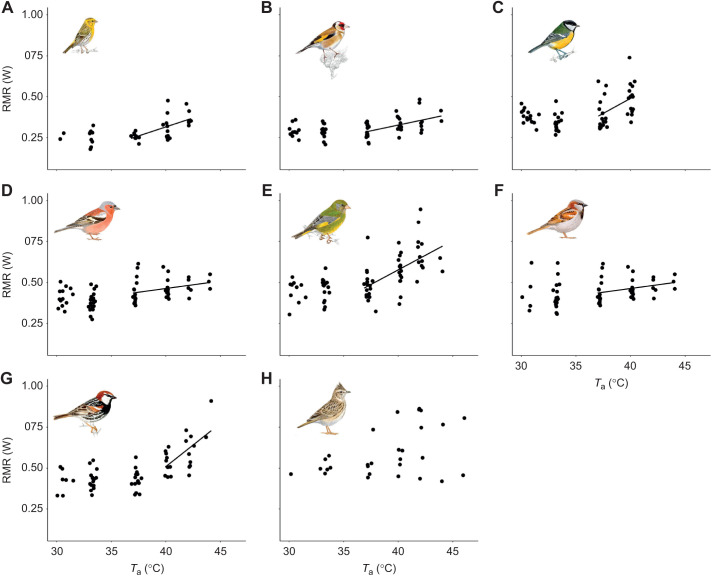
**Resting metabolic rate (RMR) as a function of air temperature (*T*_a_) in eight Mediterranean resident songbirds.** (A) Serin, (B) goldfinch, (C) great tit, (D) chaffinch, (E) greenfinch, (F) house sparrow, (G) Spanish sparrow and (H) crested lark. RMR was regressed against *T*_a_ above the upper critical temperature (*T*_uc_; see Table 1), obtaining a significant relationship in seven of the eight species. In the case of crested lark, we did not find a clear *T*_uc_. Illustrations are reproduced with the permission of Juan Varela.

RMR within the thermoneutral zone ranged between 0.26 W in the serin and 0.51 W in the crested lark (Table 1). *T*_uc_ varied from 34.16°C in the house sparrow to 37.60°C in the Spanish sparrow (Table 1). In these species, RMR increased significantly with *T*_a_ above *T*_uc_, with mass-specific slopes varying from 0.72 mW g^−1^ °C^−1^ in the house sparrow to 2.39 mW g^−1^ °C^−1^ in the great tit (Table 1, Fig. 1). In the case of the crested lark, we could not find an evident *T*_uc_, and RMR appeared to be stable through the *T*_a_ profile to which birds were exposed (Table 1, Fig. 1).

**Table 1.**
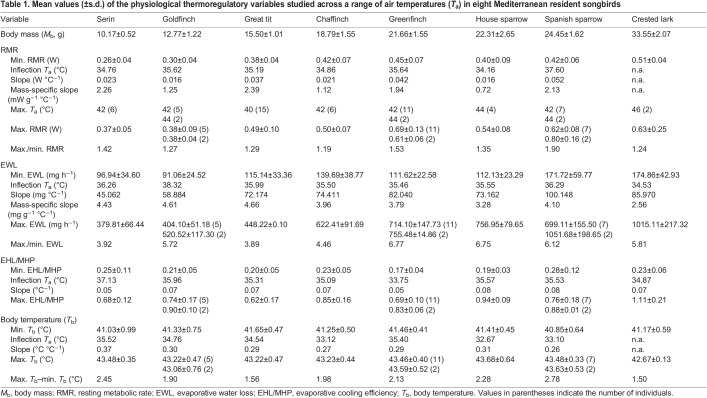
Mean values (±s.d.) of the physiological thermoregulatory variables studied across a range of air temperatures (*T*_a_) in eight Mediterranean resident songbirds

Maximum to minimum RMR ratios varied across species, from 1.19 in the chaffinch to 1.90 in the Spanish sparrow (Table 1). The interspecific analysis did not show differences across species in mass-specific RMR slope as a function of *M*_b_ (estimate=−0.03±0.05, *F*=0.28, *P*=0.617).

### Evaporative water loss

**Fig. 2.**
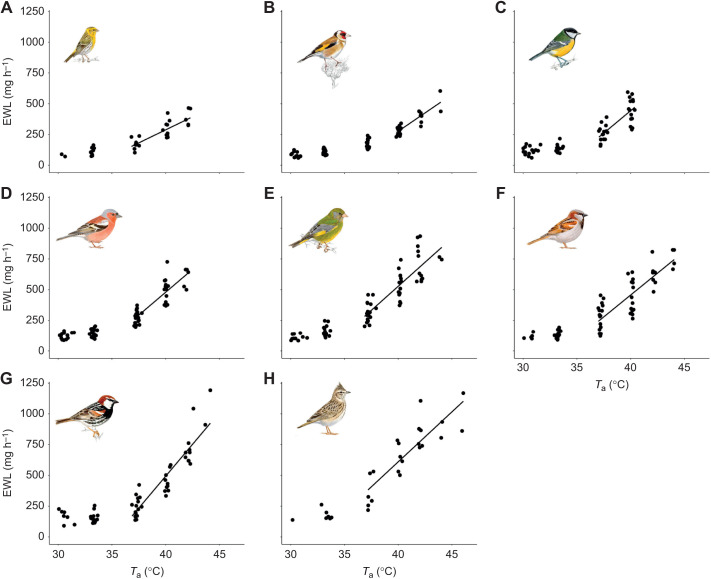
**Evaporative water loss (EWL) as a function of air temperature (*T*_a_) in eight Mediterranean resident songbirds.** (A) Serin, (B) goldfinch, (C) great tit, (D) chaffinch, (E) greenfinch, (F) house sparrow, (G) Spanish sparrow and (H) crested lark. EWL was regressed against *T*_a_ above the upper critical temperature for this variable (see Table 1), obtaining a significant relationship in all species. Illustrations are reproduced with the permission of Juan Varela.

All species showed clear inflection points in EWL, from 34.53°C in the crested lark to 38.32°C in the goldfinch (Table 1, Fig. 2). Minimum EWL varied between 91.06 mg H_2_O h^−1^ in the goldfinch and 171.72 mg H_2_O h^−1^ in the Spanish sparrow (Table 1). Above inflection points, EWL increased significantly in response to *T*_a_, with mass-specific slopes varying from 2.56 mg H_2_O g^−1^ h^−1^ °C^−1^ in the crested lark to 4.66 mg H_2_O g^−1^ h^−1^ °C^−1^ in the great tit (Table 1, Fig. 2). Maximum rates of EWL ranged from 379.81 mg H_2_O h^−1^ in the serin to 1051.68 mg H_2_O h^−1^ in the Spanish sparrow (Table 1), while evaporative scopes ranged from 3.89 in the great tit to 6.77 in the greenfinch (Table 1).

Among species, *M*_b_ had a negative influence on EWL slope (estimate=−0.086, s.e.=0.018, *F*=21.12, *P*=0.003), with smaller Mediterranean songbirds showing higher mass-specific slopes than larger ones (Table 1). However, evaporative scope did not differ in response to species *M*_b_ (estimate=0.089, s.e.=0.05, *F*=2.74, *P*=0.148).

### Evaporative cooling efficiency

**Fig. 3.**
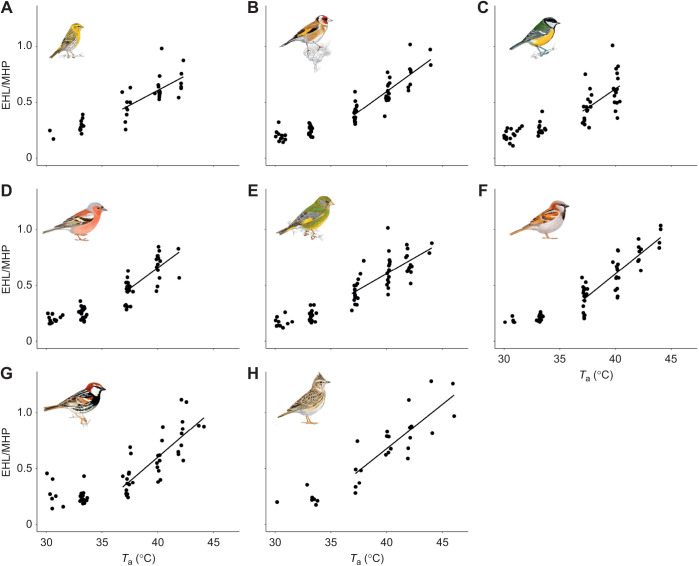
**Evaporative cooling efficiency (EHL/MHP) as a function of air temperature (*T*_a_) in eight Mediterranean resident songbirds.** (A) Serin, (B) goldfinch, (C) great tit, (D) chaffinch, (E) greenfinch, (F) house sparrow, (G) Spanish sparrow and (H) crested lark. EHL/MHP was regressed against *T*_a_ above the upper critical temperature for this variable (see Table 1), obtaining a significant relationship in all species. Illustrations are reproduced with the permission of Juan Varela.

EHL/MHP inflection points varied by 3.4°C among species, ranging from 33.53°C in the Spanish sparrow to 37.13°C in the serin (Table 1, Fig. 3). Below the inflection points, all species showed minimum EHL/MHP rates of ∼0.20 (Table 1), increasing significantly with *T*_a_ above these points at rates that varied from 0.05 to 0.08°C^−1^ (Table 1). Maximum EHL/MHP ranged from 0.62 in the great tit to 1.11 in the crested lark (Table 1).

Interspecific analyses across the eight studied species showed no effect of *M*_b_ on the EHL/MHP slope (estimate=0.001±0.001, *F*=1.49, *P*=0.267), but larger species achieved greater evaporative cooling efficiencies than smaller ones (estimate=0.016±0.01, *F*=9.81, *P*=0.020) (Table 1).

### Body temperature and heat tolerance limits

**Fig. 4.**
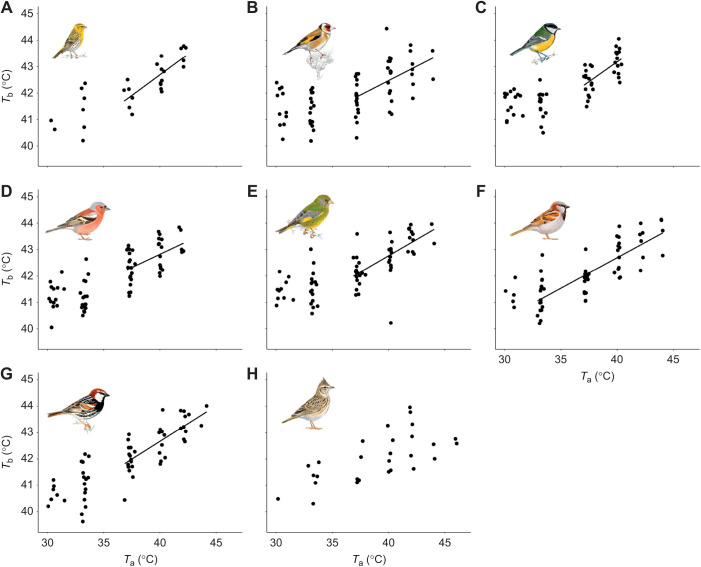
**Body temperature (*T*_b_) as a function of air temperature (*T*_a_) in eight Mediterranean resident songbirds.** (A) Serin, (B) goldfinch, (C) great tit, (D) chaffinch, (E) greenfinch, (F) house sparrow, (G) Spanish sparrow and (H) crested lark. *T*_b_ was regressed against *T*_a_ above the upper critical temperature for this variable (see Table 1), showing significant relationships in seven of the eight species. In the case of crested lark, we could not detect an inflection point for *T*_b_. Illustrations are reproduced with the permission of Juan Varela.

Normothermic *T*_b_ differed by 0.8°C across species, ranging from 40.85°C in the Spanish sparrow to 41.65°C in the great tit (Table 1). Segmented regressions revealed clear inflection points in seven of the eight species, varying from 32.67°C in the house sparrow to 35.52°C in the serin (Table 1, Fig. 4). Above inflection points, *T*_b_ increased significantly with respect to normothermic values with slopes ranging from 0.26°C °C^−1^ in the Spanish sparrow to 0.37°C °C^−1^ in the serin (Table 1). Nonetheless, the crested lark showed a different pattern, and we could not find an inflection point in *T*_b_. Along the range of *T*_a_ at which birds were exposed, they showed a steady but not significant increment in *T*_b_ (Table 1, Fig. 4).

Maximum HTL ranged from 40°C in the great tit to 46°C in the crested lark, with chaffinch and serin showing HTLs at 42°C, and goldfinch, greenfinch, Spanish sparrow and house sparrow reaching 44°C (Table 1). Most of the trials ended owing to sustained escape behaviour, with no birds reaching *T*_b_=45°C. Average *T*_b_ reached at their HTLs differed across species, from 42.67°C in the crested lark to 43.63°C in the Spanish sparrow (Table 1). Likewise, the gradient between maximum and minimum *T*_b_ values ranged from 1.50°C in the crested lark to 2.78°C in the Spanish sparrow (Table 1).

*T*_b_ slope did not differ across species with respect to *M*_b_ (estimate=−0.004±0.002, *F*=4.82, *P*=0.08). Likewise, HTL did not differ among species as a function of their *M*_b_ (estimate=0.095±0.07, *F*=6.21, *P*=0.240) and was not influenced by their evaporative scopes (estimate=0.87±0.45, *F*=6.21, *P*=0.108).

### Current and future vulnerability to high temperatures

**Fig. 5.**
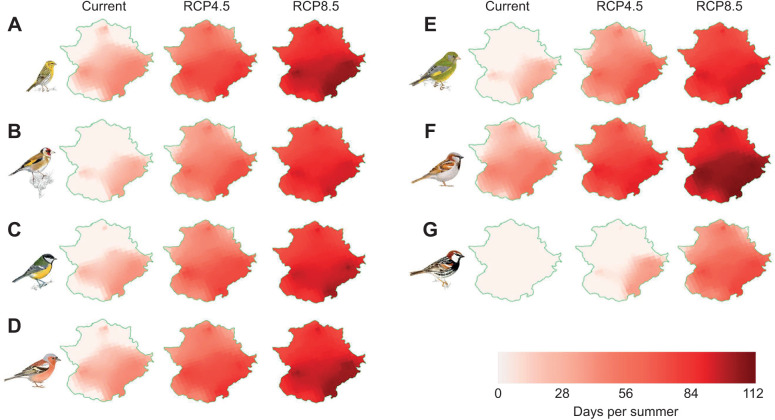
**Mean number of days per summer above the *T*_uc_ (see Table 1) experienced by the studied songbird species across Extremadura under current (2006–2021) and future (2070–2100) projected climate change scenarios (RCP4.5 and RCP8.5, respectively).** (A) Serin, (B) goldfinch, (C) great tit, (D) chaffinch, (E) greenfinch, (F) house sparrow and (G) Spanish sparrow. Crested lark could not be mapped as we could not find a clear *T*_uc_ for this species. Illustrations are reproduced with the permission of Juan Varela.

When modelling the mean number of days above *T*_uc_ across Extremadura, we found an increase for all the species studied but the crested lark (for which we could not detect a clear *T*_uc_) under projected climate change scenarios with respect to current values (Fig. 5; Table S2). At present and under future scenarios, our species showed a higher degree of vulnerability to temperature extremes along the southeastern part of the region (Fig. 5). The house sparrow showed the highest number of days per summer above *T*_uc_ (from 33.07±15.88 days at present to 98.11±8.95 days for 2070–2100 under the RCP8.5 scenario), while the Spanish sparrow showed the lowest (from 0 days at present to 54.89±14.29 days to the end of the century under the RCP8.5 scenario) (Fig. 5; Table S2).

**Fig. 6.**
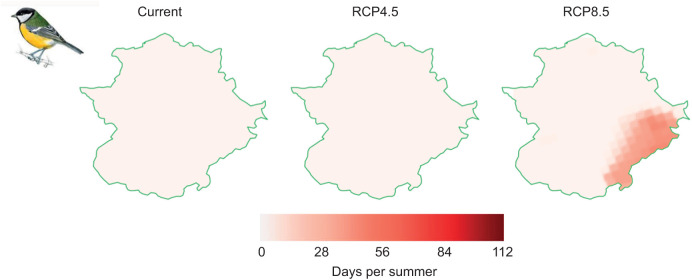
**Mean number of days per summer above HTL (see Table 1) for the great tit *Parus major* along Extremadura under current (2006–2021) and future (2070–2100) climate warming scenarios (RCP4.5 and RCP8.5, respectively).** Great tit illustration is reproduced with permission from Juan Varela.

According to model predictions, none of the eight studied species are currently experiencing days above their HTLs in Extremadura (Table S2). To the end of the century (2070–2100), only the great tit will experience days above HTL under the RCP8.5 scenario (Table S2; Fig. 6). Again, the greatest risk of lethal hyperthermia by exposure to maximum temperatures above HTL will be experienced by those populations of great tit occupying the southeast of Extremadura (see Fig. 6).

**Fig. 7.**
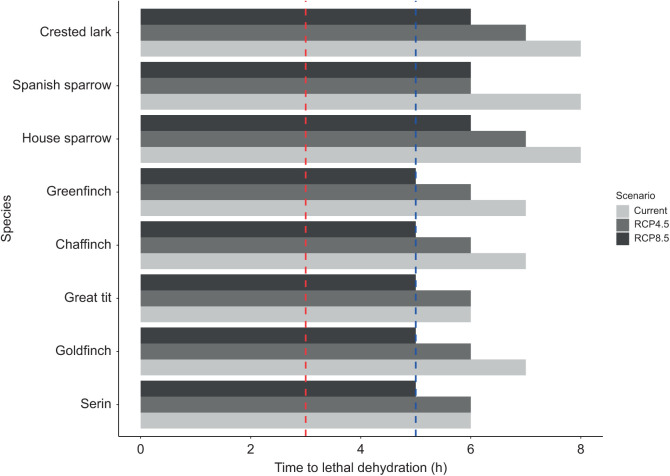
**Current (2006–2021) and future (2070–2100) times to lethal dehydration (h) during an extremely hot day under the RCP4.5 and RCP8.5 climate warming scenarios for each of the Mediterranean songbird species studied.** Dotted red line indicates a severe risk of lethal dehydration (i.e. birds losing 15% of *M*_b_ through EWL in ≤3 h), while dotted blue line indicates a moderate risk of lethal dehydration (i.e. when cumulative EWL surpassed 15% of *M*_b_ in ≤5 h).

When estimating lethal dehydration risks, none of the species studied showed moderate or severe risk of lethal dehydration during an extremely hot day under current conditions (Fig. 7). Current times to lethal dehydration during the hottest days ranged from 6 h in the serin and the great tit to up to 8 h in the house sparrow, Spanish sparrow and crested lark (Fig. 7). Similarly, times to lethal dehydration varied between 7 h in the Spanish sparrow and the crested lark to 6 h in the rest of our species under the RCP4.5 scenario, with no species experiencing moderate or severe risk. However, according to the RCP8.5 scenario, all the studied species except the crested lark and the two sparrow species will experience moderate risk of lethal dehydration during extremely hot days (Fig. 7).

## DISCUSSION

Overall, we found that small-sized Mediterranean songbirds exhibit relatively low HTL and limited cooling efficiencies, resulting in moderate risks of lethal dehydration under the most severe climate change scenario. The HTLs of our small-sized species ranged from 40 to 46°C. These values are among those reported for tropical (39–45.5°C) and temperate North American songbirds (39.6–45.9°C) (Pollock et al., 2020), as well as for an Arctic species (43°C; O'Connor et al., 2021). To some degree they also overlapped with the HTLs of songbirds from humid lowland and cool montane sites from South Africa (43.3–50.9°C) (Freeman et al., 2022). As predicted, however, Mediterranean species showed HTLs substantially lower than those reached by similar-sized songbirds from deserts and arid zones (46–54°C) (Whitfield et al., 2015; McKechnie et al., 2017; Smith et al., 2017; Smit et al., 2018; Kemp and McKechnie, 2019; Czenze et al., 2020). This limited heat tolerance among our species with respect to arid-zone species could result from greater endogenous heat production (higher mass-specific RMR slopes and *T*_b_ slopes) and limited evaporative heat dissipation capacity (lower evaporative scopes), as discussed below.

Mediterranean songbirds showed *T*_uc_ values similar to those found across songbirds from arid zones (Whitfield et al., 2015; McKechnie et al., 2017; Smith et al., 2017; Czenze et al., 2020), as well as from tropical and temperate zones from North America (Pollock et al., 2020) or those from lowland and montane sites in South Africa (Freeman et al., 2022). However, the *T*_uc_ values among the Mediterranean species studied here were above that reported for the Arctic songbird (O'Connor et al., 2021). Our results, therefore, highlight the limitations of using *T*_uc_ as a proxy of species' thermal tolerance in predictive models of the vulnerability of species to climate change (as in Khaliq et al., 2014), as HTLs are well above *T*_uc_ and there is not a clear relationship between both traits (Mitchell et al., 2018). Among our species, all but the crested lark showed a clear *T*_uc_, which differed across species. This indicates that some species show an earlier onset of active heat dissipation than others. Most notably, *T*_uc_ differed greatly between house and Spanish sparrows even though they reached the same HTL. The more urban house sparrows showed a lower *T*_uc_ value than Spanish sparrows, which live in more open habitats. Furthermore, the absence of a clear *T*_uc_ in the crested lark points in the same direction; that is, an adaptation of open-landscape species to forage under greater heat loads. This would enable them to postpone the beginning of active heat dissipation and thus extend foraging activity.

Our species displayed 1.4-fold increases in RMR respect to thermoneutral values at their HTLs, a value within the range shown by arid-zone songbirds (Whitfield et al., 2015; McKechnie et al., 2017; Smith et al., 2017; Smit et al., 2018; Kemp and McKechnie, 2019; Czenze et al., 2020), South African lowland and montane songbirds (Freeman et al., 2022), and the Arctic snow bunting, *Plectrophenax nivalis* (O'Connor et al., 2021). However, the mass-specific RMR slopes of our songbirds were higher than those of arid-zone species, but similar to those of birds from lowland and montane sites in South Africa (Table 2). This suggests that above *T*_uc_, our species generate a greater amount of endogenous heat loads, which could partly explain why they achieved lower HTLs than similar-sized songbirds from deserts and arid zones. Nevertheless, our species also showed greater mass-specific RMR slopes than the Arctic snow bunting (O'Connor et al., 2021) (Table 2), probably owing to the lower *T*_uc_ shown by the latter. This implies earlier accumulation of metabolic heat, but at a lower rate compared with our species, which can postpone the beginning of panting at higher *T*_a_ but results in faster RMR increases.

**Table 2.**
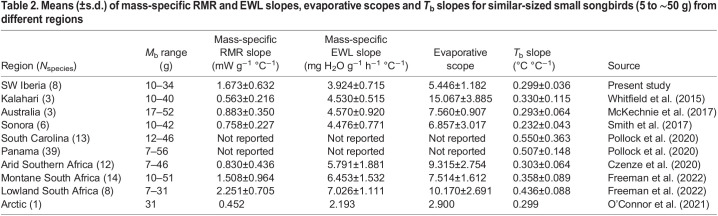
Means (±s.d.) of mass-specific RMR and EWL slopes, evaporative scopes and *T*_b_ slopes for similar-sized small songbirds (5 to ∼50 g) from different regions

Above thermoneutrality, our songbirds showed marked increases in EWL starting when the *T*_b_–*T*_a_ gradient was still wide enough (∼4.34°C). Snow bunting showed a similar pattern (O'Connor et al., 2021), while arid-zone songbirds delayed EWL inflection points (∼37°C to 45°C) until *T*_a_ approached or exceeded *T*_b_ (Whitfield et al., 2015; McKechnie et al., 2017; Smith et al., 2017; Czenze et al., 2020). This could be viewed as an adaptation of the arid-zone species for water conservation, while our species generally have access to drinking water and can thus compensate EWL. The studied species had mass-specific EWL slopes similar to those of arid-zones species, but lower than South African species from lowland and montane sites (Table 2). However, Mediterranean songbirds achieved lower evaporative scopes than Southern African songbirds inhabiting either arid or humid habitats (Whitfield et al., 2015; Czenze et al., 2020; Freeman et al., 2022), but similar to those from arid Australia or North America (McKechnie et al., 2017; Smith et al., 2017) and from a montane site in South Africa (Freeman et al., 2022) (Table 2). On the contrary, Mediterranean songbirds showed greater evaporative scopes than snow buntings (O'Connor et al., 2021) (Table 2). This limited evaporative scope among our Mediterranean songbirds compromises their capacity to dissipate endogenous heat loads, thus resulting in limited cooling efficiency and lower HTLs. Besides, among our species, smaller ones showed greater mass-specific EWL slopes, thus resulting in greater risks of lethal dehydration as discussed below.

**Fig. 8.**
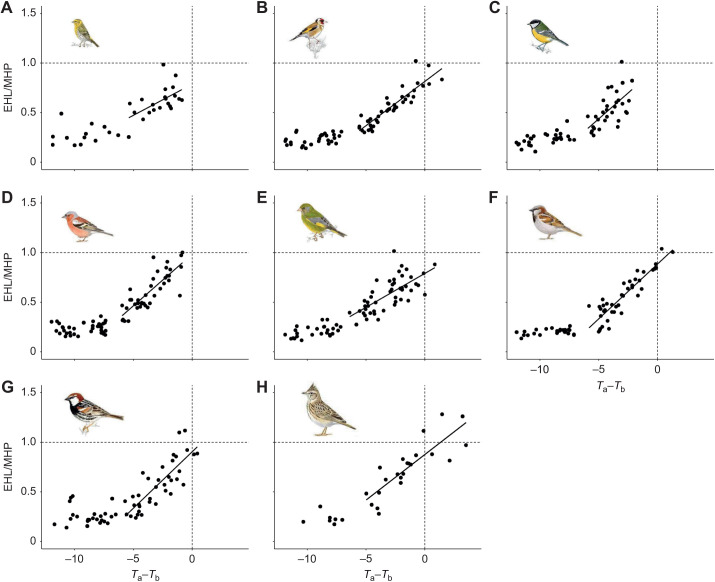
**Evaporative cooling efficiency (EHL/MHP) as a function of the gradient between air temperature and body temperature (*T*_a_–*T*_b_) for each of the Mediterranean songbirds studied.** (A) Serin, (B) goldfinch, (C) great tit, (D) chaffinch, (E) greenfinch, (F) house sparrow, (G) Spanish sparrow and (H) crested lark. EHL/MHP was regressed against *T*_a_–*T*_b_ above the upper critical threshold for this variable (which varies from −7.75°C in the greenfinch to −5.42°C in the house sparrow). Dashed lines indicate the intersection at which EHL/MHP achieve values close to 1 (horizontal dashed line) when *T*_a_–*T*_b_ is 0 (vertical dashed line). Illustrations are reproduced with the permission of Juan Varela.

Indeed, the maximum evaporative cooling efficiencies of Mediterranean songbirds were at the lower end of those reported across the clade. This confirms previous findings showing a limited cooling efficiency (EHL/MHP) in a Mediterranean songbird, the great tit (Playà-Montmany et al., 2021). Among the species studied here, larger-bodied songbirds achieved greater EHL/MHP, supporting the notion that larger-sized species deal better with heat than smaller species (reviewed by McKechnie et al., 2021a,b). Nonetheless, only the crested lark (the largest species in our dataset) dissipated all the heat metabolically produced to achieve the maximum evaporative cooling efficiency (EHL/MHP=1.11); yet for all the species, EHL/MHP approached 1 when *T*_a_ was similar to *T*_b_ (Fig. 8).

Above inflection points, *T*_b_ increased with *T*_a_, suggesting that facultative hyperthermia is a common thermoregulatory response used by Mediterranean songbirds. This increase in *T*_b_ allowed birds to maintain a favourable gradient (*T*_b_>*T*_a_), thus permitting passive heat dissipation and saving body water by delaying EWL (Tieleman and Williams, 1999; Gerson et al., 2019). In a global context, *T*_b_ slopes in response to increasing *T*_a_ were similar to those of arid-zone, South African montane and Arctic songbirds (Whitfield et al., 2015; McKechnie et al., 2017; Smith et al., 2017; Czenze et al., 2020; O'Connor et al., 2021; Freeman et al., 2022), but lower than those found in North American temperate and tropical species, and in those from a lowland South African site (Pollock et al., 2020; Freeman et al., 2022) (Table 2). However, our species started to increase *T*_b_ at lower *T*_a_ than arid-zone songbirds (Whitfield et al., 2015; McKechnie et al., 2017; Smith et al., 2017; Czenze et al., 2020), resulting in earlier increases in endogenous heat loads. This, together with higher mass-specific RMR slopes, lower evaporative scopes and lower evaporative cooling efficiencies, could explain the lower HTLs achieved by our species compared with arid-zone species.

At their HTLs, Mediterranean songbirds achieved mean maximum *T*_b_ values (42.7–43.7°C) similar to those of snow buntings (43.5°C), but lower than those of arid-zone species (43.5–45.3°C; Whitfield et al., 2015; McKechnie et al., 2017; Smith et al., 2017) or South African songbirds from a lowland site and a montane site (44.5–48°C; Freeman et al., 2022). To ensure that birds remain calm at *T*_a_ near lethal hyperthermia thresholds (∼45°C) and thus elicit meaningful maximum *T*_b_ values, researchers often use relatively high flow rates (e.g. Whitfield et al., 2015; McKechnie et al., 2017; Freeman et al., 2022). Thus, it could be argued that the lower *T*_b_ values observed in our species were the result of the relatively low flow rates used in this study. We set flow rates that ensured that birds remained calm and chamber humidity low during the heat tolerance assessments while permitting the O_2_ signal to remain detectable by the analyzer. We are confident that we pushed birds near their upper heat tolerance thresholds, as we recorded instances of loss of righting response at *T*_b_ as low as 43.3°C in a crested lark or 43.7–44.2°C in great tits during heat tolerance trials. Nonetheless, we cannot rule out that the lower *T*_b_ values observed here were partly caused by the different methods used to measure *T*_b_. We measured *T*_b_ with subcutaneously implanted PIT tags to minimize harm to birds, while previous studies measured core *T*_b_ by implanting PIT tags in the abdominal cavity or in the cloaca, or by inserting thermocouples in the cloaca (e.g. Whitfield et al., 2015; McKechnie et al., 2017; Pollock et al., 2020; O'Connor et al., 2021). For instance, subcutaneous *T*_b_ measured in the interscapular region of snow buntings was up to 0.6±1.0°C lower than that measured in the cloaca (see supplementary material in O'Connor et al., 2021). Hence, Mediterranean songbirds could have reached slightly higher *T*_b_ values than reported here.

Among Mediterranean songbirds, the crested lark – which inhabits open landscapes and usually faces high solar radiation loads and operative temperatures that surpass *T*_b_ – achieved the highest HTL (46°C), a value identical to that recorded for two arid-zone songbirds (the yellow-plumed honeyeater *Lichenostomus ornatus* and the Orange River white-eye *Zosterops pallidus*) (McKechnie et al., 2017; Czenze et al., 2020). In contrast, the great tit – mainly a forest dweller – only tolerated a maximum of 40°C during the trials, suggesting that this species probably resorts to behavioural thermoregulation to avoid lethal hyperthermia during heat events, as this temperature is often exceeded every summer in our study area. These differences among species and the relatively low HTLs of Mediterranean songbirds suggest a strong influence of the natural temperature regime experienced by the species on their heat tolerance, with those usually facing environmental temperatures approaching or exceeding *T*_b_ reaching greater HTLs (as reviewed in McKechnie and Wolf, 2019; Freeman et al., 2022).

### Current and future vulnerability to high temperatures

Regardless of the period and the climate change scenario considered, maximum environmental temperatures experienced during summer in Extremadura forced Mediterranean songbirds to engage in physiological thermoregulation. Among species, the mean number of days above *T*_uc_ differed markedly, pointing to different risks of sublethal chronic effects of heat exposure. Those species mainly foraging in open landscapes, such as the Spanish sparrow, the greenfinch and the goldfinch, showed a lower number of days per summer in which they have to resort active thermoregulation, whereas urban (the house sparrow) and mainly forest songbirds (the great tit and the chaffinch), as well as our smallest species (the serin), experienced a greater mean number of days above *T*_uc_ (Table S2). Therefore, the latter species could be more prone to deleterious fitness effects of repeated exposure to environmental temperatures surpassing their *T*_uc_ (e.g. through trade-offs and constraints that thermoregulatory mechanisms may induce) (Du Plessis et al., 2012; Cunningham et al., 2021). Yet, only the great tit (mainly a forest dweller) will experience direct risk of heat-related mortality across Extremadura. To the end of the century, climatic models forecast that great tits will experience on average 5 days per summer in which *T*_max_ will surpass its HTL in the southeast of the region under the RCP8.5 warming scenario.

Mediterranean songbirds might reduce lethal dehydration risk by combining the use of thermally buffered microhabitats during the hottest part of the day (such as shaded places near water streams, shade from shrubs, or tree hollows) and regularly drinking to sustain high EWL rates needed to avoid lethal hyperthermia. Despite this, the forecasted temperature increase by the end of the century indicates that all of the study species (except the crested lark and the house and Spanish sparrows) will experience a moderate risk of lethal dehydration under the RCP8.5 warming scenario. This highlighted that across our studied species, smaller ones are more prone to experience dehydration risks as their higher mass-specific EWL slopes result in faster loss of body water, similar to that reported for arid-zone species (McKechnie and Wolf, 2010). Furthermore, it is important to note that the EWL rates employed to calculate lethal dehydration times come from resting individuals under laboratory conditions (i.e. overlooking the effect of solar radiation or activity). In the wild, these rates could be substantially higher owing to locomotor activity, heat increment of feeding (González-Medina et al., 2020), or higher environmental heat loads. This, combined with the predicted increase in heavy droughts along the Mediterranean Basin (IPCC, 2021), could lead to shorter times to lethal dehydration than predicted among Mediterranean songbirds. In turn, this might result in severe risk of lethal dehydration as forecasted for several arid-zone songbirds (McKechnie and Wolf, 2010; Albright et al., 2017; Conradie et al., 2020).

### Conclusions

Our findings indicate that Mediterranean songbirds are more sensitive to high-temperature extremes than arid-zone and desert songbirds. Their limited cooling efficiencies are likely due to their relatively low evaporative scopes, which cannot compensate for the endogenous heat loads (RMR and *T*_b_ increases) in response to heat. Although the studied species are not currently experiencing lethal dehydration risks, they all are experiencing several summer days in which their thermoregulatory capacities are challenged. This will be exacerbated under predicted future warming scenarios, which will increase the risks of lethal dehydration during heat waves and, ultimately, threaten the persistence of songbird species in southwestern Spain and other Mediterranean regions that are warming rapidly (IPCC, 2021).

## Supplementary Material

10.1242/jexbio.244848_sup1 Supplementary information
