# Supplementary material for: Heat tolerance limits of Mediterranean songbirds and their current and future vulnerabilities to temperature extremes
Source: J Exp Biol. 2022 Dec 9;225(23):jeb244848. doi: 10.1242/jeb.244848 (PMC9789400; doi:10.1242/jeb.244848)
Supplement: Supplementary information [file jexbio-225-244848-s1.pdf]

SUPPLEMENTARY MATERIALS & METHODS

Study area

This study took place in Badajoz (38º51’ N, 6º58’ W), which is part of Extremadura autonomous region, southwestern Spain, and is located in the western end of the Mediterranean Basin (Fig. S1).

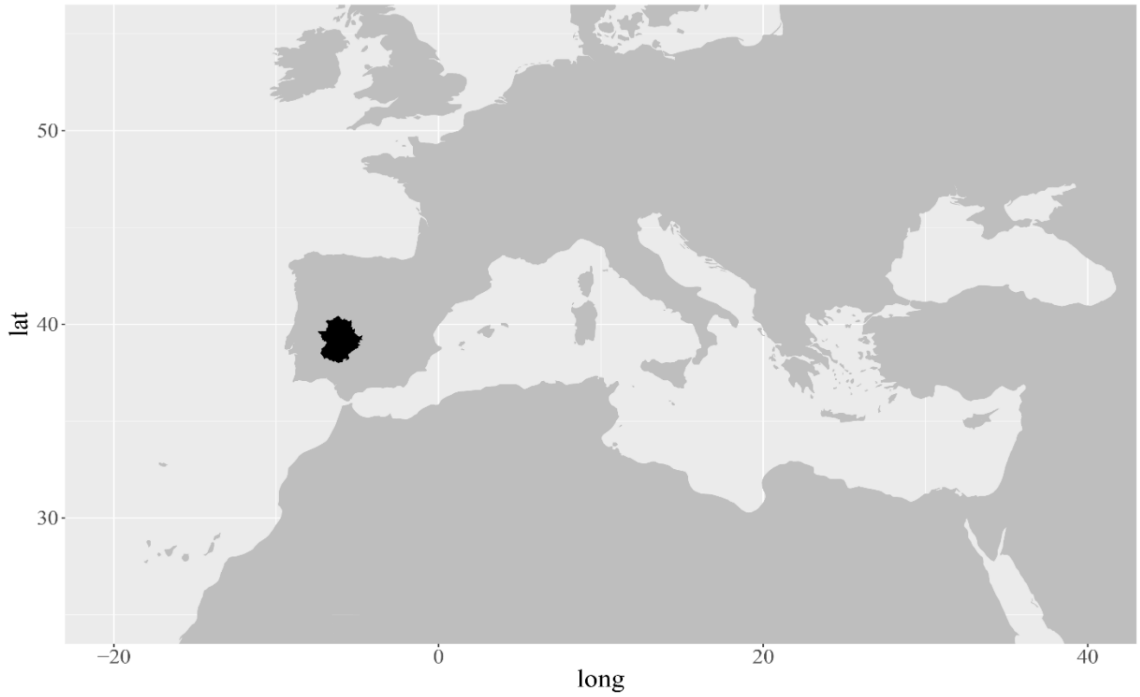

Fig. S1. Map showing the Mediterranean Basin, with Extremadura highlighted in black.

Climatological data

During the period 1993-2019, the analysis of climatological data from Badajoz airport weather station (Spanish Meteorology Agency) showed a significant increase in the number of hot days (i.e., those in which maximum ambient temperature reached or surpassed 35ºC) during summer ($\beta = 1.01$; $p = < 0.001$) (Fig. S2).

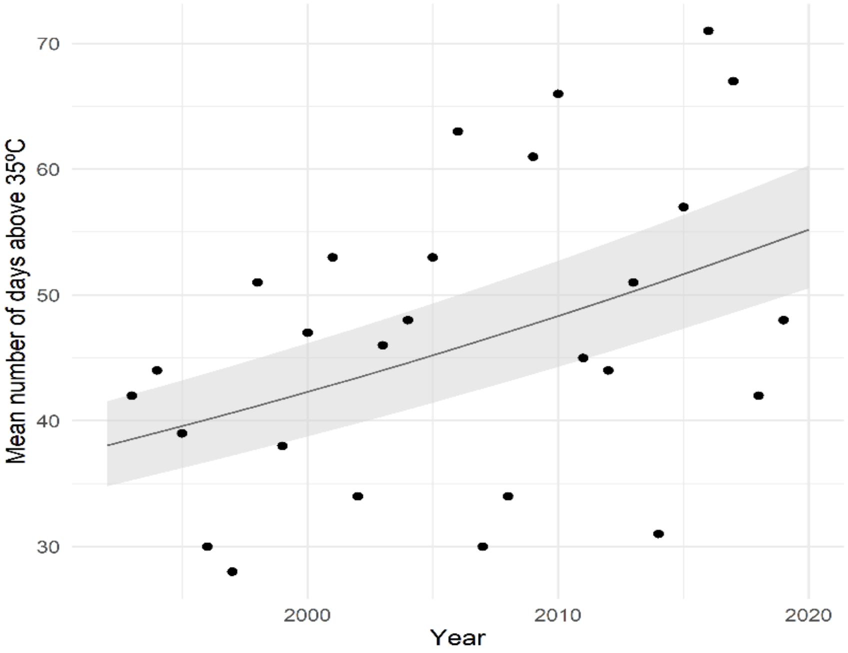

Fig. S2. Number of hot days during summer (≥ 35ºC) along the 1993-2019 period in the study area.

Phylogenetic interspecific comparisons

We tested for possible interspecific differences in thermoregulatory traits among Mediterranean songbirds due to phylogenetic relatedness and body size. To do so, we employed the R package 'caper' (Orme et al. 2018) to adjust phylogenetic generalized least squares models to different thermoregulatory traits (mass-specific RMR slope, mass-specific EWL slope, evaporative scope, EHL/MHP slope, maximum EHL/MHP, T_b slope, and HTL), with mean body mass (M_b) of each species as predictor variable. We accounted for phylogeny effect by including a phylogenetic consensus tree, which we

Journal of Experimental Biology • Supplementary information

derived from 10,000 trees with different topologies obtained from Bird Tree project using 'Hackett All Species' as backbone (Jetz et al. 2012). When lambda (λ) = 0 the covariance between species is null, which indicates that a non-phylogenetic regression is more appropriate for data. On the contrary, λ = 1 denote a strong phylogenetic signal in data.

We obtained null phylogenetic signal for each thermoregulatory trait tested, as λ= 0 (Table S1). Therefore, we opted to analyse our data using non-phylogenetic generalized linear models.

Table S1. Statistics from phylogenetic generalized least squares models adjusted to different thermal traits of Mediterranean songbirds. In each model, λ equals to zero and indicates and absence of phylogenetic signal in the data.

Model		Estimate	s.e	t-value	p-value	λ
RMR_{slope} ~ M_b						0.000
	Intercept	0.005	0.02	0.28	0.793	
	M_b	0.001	0.001	1.32	0.244	
EWL_{slope} ~ M_b						0.000
	Intercept	18.725	10.95	1.71	0.148	
	M_b	2.983	0.59	5.07	0.004	
Evaporative scope ~ M_b						0.000
	Intercept	2.359	1.42	1.67	0.157	
	M_b	0.168	0.08	2.21	0.078	
EHL/MHP_{slope} ~ M_b						0.000
	Intercept	0.047	0.02	2.72	0.042	
	M_b	0.001	0.001	1.21	0.280	
Max EHL/MHP ~ M_b						0.000
	Intercept	0.587	0.15	3.82	0.012	
	M_b	0.013	0.01	1.53	0.186	
$T_{b\ slope}$~ M_b						0.000
	Intercept	0.383	0.04	9.58	<0.001	
	M_b	-0.005	0.002	-2.19	0.079	
HTL ~ M_b + Evaporative scope						0.000
	Intercept	37.111	1.37	27.11	<0.001	
	M_b	-0.090	0.08	-1.09	0.337	
	Evaporative scope	1.370	0.35	3.95	0.017	

Future climate warming projections

We downloaded future daily maximum environmental temperature projections from Coupled Model Intercomparison Project 5 (CMIP5) for climate warming scenarios RCP4.5 and RCP8.5 from the Spanish Climate Change Office' project *AdapteCCa* (https://www.adaptecca.es/). This dataset contained projections from the following 16 models from EURO-CORDEX (Jacob et al. 2014): CNRM-CERFACS-CNRM-

CM5_r1i1p1_CLMcom-CCLM4-8-17_v1; CNRM-CERFACS-CNRM-CM5_r1i1p1_CNRM-ALADIN53_v1; CNRM-CERFACS-CNRM-CM5_r1i1p1_SMHI-RCA4_v1; ICHEC-EC-EARTH_r12i1p1_CLMcom-CCLM4-8-17_v1; ICHEC-EC-EARTH_r12i1p1_SMHI-RCA4_v1; ICHEC-EC-EARTH_r1i1p1_KNMI-RACMO22E_v1; ICHEC-EC-EARTH_r3i1p1_DMI-HIRHAM5_v1; IPSL-IPSL-CM5A-MR_r1i1p1_IPSL-INERIS-WRF331F_v1; IPSL-IPSL-CM5A-MR_r1i1p1_SMHI-RCA4_v1; MOHC-HadGEM2-ES_r1i1p1_CLMcom-CCLM4-8-17_v1; MOHC-HadGEM2-ES_r1i1p1_KNMI-RACMO22E_v2; MOHC-HadGEM2-ES_r1i1p1_SMHI-RCA4_v1; MPI-M-MPI-ESM-LR_r1i1p1_CLMcom-CCLM4-8-17_v1; MPI-M-MPI-ESM-LR_r1i1p1_SMHI-RCA4_v1a; MPI-M-MPI-ESM-LR_r2i1p1_MPI-CSC-REMO2009_v1; NCC-NorESM1-M_r1i1p1_DMI-HIRHAM5_v2.

Hourly profiles of maximum ambient temperature during heatwaves

We calculated hourly maximum temperature profile during an extremely hot day by averaging hourly values from the hottest 10 days recorded in our study area during the last 20 years (namely 10th of August 2012, 6th of September 2016, 16th and 17th of June 2017, 13th of July 2017, 2nd, 3rd, 4th, 5th and 6th of August 2018) (Fig. S3). To this current hourly profile, we added 1.8 and 3.7 ºC respectively to represent climate warming scenarios RCP4.5 and RCP8.5, respectively (Fig. S3).

Journal of Experimental Biology • Supplementary information

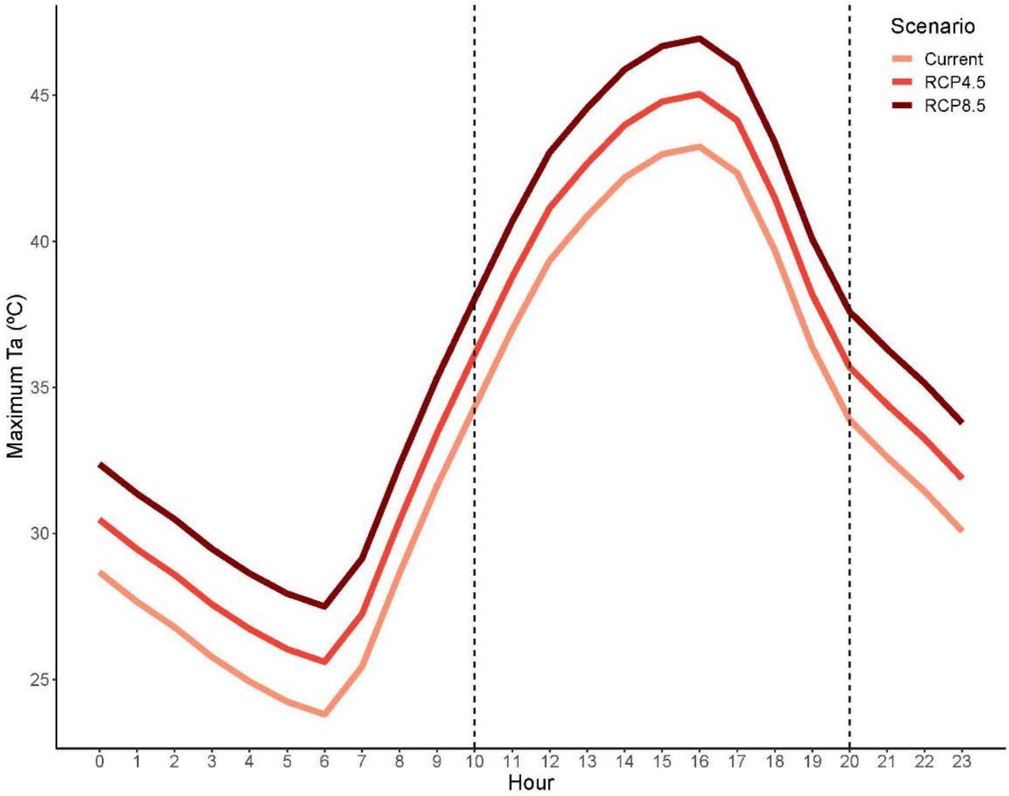

Fig. S3. Daily hourly profile of ambient temperature during extremely hot days in the study area (data obtained from Badajoz Airport weather station, Spanish Meteorology Agency) at present and in the future (2070-2100) under two different climate warming scenarios (RCP4.5 and RCP8.5). Dotted lines denoted the time period during which lethal dehydration risk was calculated for the studied Mediterranean songbirds.

RESULTS

Current and future vulnerabilities of Mediterranean songbirds

We calculated the mean number of days in which maximum environmental temperature is above the upper critical temperature (T_{uc}) and the heat tolerance limit (HTL) for each species along Extremadura under current and future climate warming scenarios (RCP4.5 and RCP8.5) (Table S2).

Journal of Experimental Biology • Supplementary information

Table S2. Mean number of days (± S.D.) per summer (June to September) above the Tuc and above the HTL for each species at current (2006-2021) and under future (2070-2100) climate warming scenarios along Extremadura. We could not calculate days above Tuc for the crested lark as we could not identify a clear Tuc for this species.

	Days above T_{uc}			Days above HTL		
	**Current**	**RCP4.5**	**RCP8.5**	**Current**	**RCP4.5**	**RCP8.5**
Serin	20.38 ± 16.88	63.64 ± 13.76	91.38 ± 8.77	0	0	0
Goldfinch	7.18 ± 11.44	47.51 ± 15.26	82.69 ± 7.37	0	0	0
Great tit	13.09 ± 14.89	55.87 ± 14.33	86.78 ± 7.65	0	0	4.94 ± 11.06
Chaffinch	18.55 ± 16.74	61.90 ± 13.92	90.24 ± 8.56	0	0	0
Greenfinch	6.94 ± 11.27	47.13 ± 15.40	82.46 ± 7.39	0	0	0
House sparrow	33.07 ± 15.88	73.70 ± 11.81	98.11 ± 8.95	0	0	0
Spanish sparrow	0	7.63 ± 13.03	54.89 ± 14.29	0	0	0
Crested lark	n.a.	n.a.	n.a.	0	0	0

REFERENCES

Jacob, D., Petersen, J., Eggert, B., Alias, A., Christensen, O.B., Bouwer, L.M., et al. (2014). EURO-CORDEX: a new high-resolution climate change projections for European impact research. *Reg. Environ.* **14**(2), 563-568. https://doi.org/10.1007/s10113-013-0499-2

Jetz, W., Thomas, G.H., Joy, J.B., Hartmann, K., and Mooers, A.O. (2012). The global diversity of birds in space and time. *Nature* **491**, 444-448. https://doi.org/10.1038/nature11631

Orme, D., Freckleton, R., Thomas, G., Petzoldt, T., Fritz, S., Isaac, N., and Pearse, W. (2018). caper: comparative analyses of phylogenetics and evolution in R. R package version 1.0.1.

Journal of Experimental Biology • Supplementary information
